# SKN-1 activation during infection of *Caenorhabditis elegans* requires CDC-48 and endoplasmic reticulum proteostasis

**DOI:** 10.1093/genetics/iyae131

**Published:** 2024-08-21

**Authors:** Carolaing Gabaldón, Ozgur Karakuzu, Danielle A Garsin

**Affiliations:** Department of Microbiology and Molecular Genetics, The University of Texas Health Science Center at Houston, Houston, TX 77030, USA; Department of Microbiology and Molecular Genetics, The University of Texas Health Science Center at Houston, Houston, TX 77030, USA; Department of Microbiology and Molecular Genetics, The University of Texas Health Science Center at Houston, Houston, TX 77030, USA

**Keywords:** *Caenorhabditis elegans*, CDC-48, SKN-1, Nrf, infection, stress response

## Abstract

During challenge of *Caenorhabditis elegans* with human bacterial pathogens such as *Pseudomonas aeruginosa* and *Enterococcus faecalis*, the elicited host response can be damaging if not properly controlled. The activation of Nrf (nuclear factor erythroid-related factor)/CNC (Cap-n-collar) transcriptional regulators modulates the response by upregulating genes that neutralize damaging molecules and promote repair processes. Activation of the *C. elegans* Nrf ortholog, SKN-1, is tightly controlled by a myriad of regulatory mechanisms, but a central feature is an activating phosphorylation accomplished by the p38 mitogen-activated kinase (MAPK) cascade. In this work, loss of CDC-48, an AAA+ ATPase, was observed to severely compromise SKN-1 activation on pathogen and we sought to understand the mechanism. CDC-48 is part of the endoplasmic reticulum (ER)-associated degradation (ERAD) complex where it functions as a remodeling chaperone enabling the translocation of proteins from the ER to the cytoplasm for degradation by the proteosome. Interestingly, one of the proteins retrotranslocated by ERAD, a process necessary for its activation, is SKN-1A, the ER isoform of SKN-1. However, we discovered that SKN-1A is not activated by pathogen exposure in marked contrast to the cytoplasmic-associated isoform SKN-1C. Rather, loss of CDC-48 blocks the antioxidant response normally orchestrated by SKN-1C by strongly inducing the unfolded protein response (UPR^ER^). The data are consistent with the model of these 2 pathways being mutually inhibitory and support the emerging paradigm in the field of coordinated cooperation between different stress responses.

## Introduction

Inflammation is an important and necessary aspect of the immune response required for overcoming infectious agents. However, inappropriate or prolonged inflammation can contribute to poor outcomes highlighting the necessity for careful control of this response. One source of inflammation is reactive oxygen species (ROS) produced both as a by-product of cellular metabolism and by purposeful generation. We previously discovered that an NADPH oxidase, BLI-3, mediates ROS generation in *Caenorhabditis elegans* in response to infection by human bacterial pathogens that include *Enterococcus faecalis* and *Pseudomonas aeruginosa* (Chávez *et al.* 2009; Garsin 2023). While the response is generally protective and enhances survival, too much ROS can cause cellular damage and, not surprisingly, mechanisms are in place to limit ROS accumulation and to repair damage.

One component that serves this role in *C. elegans* is SKN-1, a member of the Nrf (nuclear factor erythroid 2-related factor)/CNC (Cap-n-collar) family of transcriptional regulators that upregulates protective genes (reviewed by Blackwell *et al.* 2015). We and others previously showed that SKN-1 is activated upon infection with pathogen in a manner dependent on BLI-3 (Hoeven *et al.* 2011; Papp *et al.* 2012; van der Hoeven *et al.* 2012). Moreover, the activation is beneficial and animals lacking BLI-3 or SKN-1 are more susceptible to infection (Hoeven *et al.* 2011; Papp *et al.* 2012). Likewise, a mammalian SKN-1 ortholog, Nrf2, was discovered to have protective properties against a diverse array of infections (Thimmulappa *et al.* 2006; Segal *et al.* 2010; Gomez *et al.* 2016; Chiu *et al.* 2017).

In *C. elegans*, 4 different isoforms of SKN-1 are encoded, only 3 of which are expressed—*skn-1a*, *skn-1b*, and *skn-1c* (reviewed by Blackwell *et al.* 2015). The SKN-1C is an isoform found mainly in the cytoplasm of the intestinal cells. However, it was observed to translocate to the nuclei under activating stress conditions that include oxidant exposure and exposure to pathogen (An and Blackwell 2003; Hoeven *et al.* 2011). SKN-1B is mainly found in certain neurons including the ASI neurons where it is constitutively localized to the nuclei (An and Blackwell 2003). SKN-1A is found in most cell types, including the intestine, but it contains a transmembrane domain lacking in the other isoforms that cause it to associate with the ER and mitochondrial membranes (Lehrbach and Ruvkun 2016). SKN-1A is initially translated into the ER but then is promptly retrotranslocated to the cytosolic side of the membrane by the endoplasmic reticulum (ER)-associated degradation (ERAD) complex where it undergoes rapid degradation by the proteosome. However, in cells experiencing a loss of proteostasis, SKN-1A escapes proteosomal degradation and instead undergoes a single cleavage event to generate its transcriptionally active form (Lehrbach and Ruvkun 2016). Interestingly, it has been recognized that SKN-1 supports proteostasis and can upregulate proteosome genes in a manner likely dependent on the SKN-1A isoform (Glover-Cutter *et al.* 2013; Hourihan *et al.* 2016; Lehrbach and Ruvkun 2016; Castillo-Quan *et al.* 2023). However, whether SKN-1A, specifically, is activated during pathogen exposure has not been investigated to our knowledge.

While screening for genes that affect activation of SKN-1 upon exposure to pathogen (Wu *et al.* 2021), we observed that targeting *cdc-48* by RNAi resulted in a profound loss of SKN-1 activity. CDC-48/VCP/p97 are members of the AAA+ family of ATPases that form monomeric hexamers and generally have substrate remodeling activity (reviewed by Meyer and Weihl 2014; Barthelme and Sauer 2016). The chaperone-like activity of these proteins contributes to a number of important cellular functions including association with the ERAD complex that retrotranslocates misfolded proteins from the ER lumen to the cytoplasm where they are degraded by the proteasome (reviewed by Meyer and Weihl 2014; Barthelme and Sauer 2016). In addition to the specific effects on SKN-1A, mentioned above, loss of CDC-48 would be predicted to generally block protein retrotranslocation and cause a loss of proteostasis that could activate the unfolded protein response in the ER (UPR^ER^). Interestingly, previous work demonstrated that activating the UPR^ER^ blocks SKN-1's protective response to oxidative stress (Hourihan *et al.* 2016).

In this study, we sought to elucidate the requirement for CDC-48 in inducing SKN-1's protective activity on pathogen. Based on the previous work mentioned above, 2 possible mechanisms of action were examined. First, we explored whether CDC-48 was mediating its effects through the SKN-1A isoform, which is known to require the ERAD complex for activation, of which CDC-48 is a part (Lehrbach and Ruvkun 2016; Castillo-Quan *et al.* 2023). Second, we investigated whether loss of CDC-48 activates the UPR^ER^ and if the activation of this stress response is indirectly blocking the antioxidant response of SKN-1, associated with the SKN-1C isoform (Hourihan *et al.* 2016). The results provided support for the second mechanism. It was found that the SKN-1A isoform was not activated by exposure to *E. faecalis* or *P. aeruginosa*. However, loss of CDC-48 induced the UPR^ER^ and suppressed SKN-1C and the antioxidant response normally upregulated on pathogen by affecting the upstream signaling pathway.

## Materials and methods

### Growth and maintenance of *C. elegans*

The *C. elegans* and bacterial strains used in this study are listed in Supplementary Table 1. To maintain *C. elegans* strains, the worms were cultured on nematode growth (NG) agar plates (as previously described; Hope 1999) with *Escherichia coli* strain OP50 at 20°C. Briefly, OP50 was cultured overnight from glycerol stocks on Luria-Bertani (LB) plates at 37°C. Following this, a bacterial sample was used to inoculate LB liquid medium and grown overnight at 210 rpm at 37°C. The medium containing bacteria was centrifuged and concentrated 3 times. Five hundred microliters to one milliliter of bacteria was used to inoculate NG agar plates, which were left to dry and then utilized as food for the worms. For experiments requiring synchronized animals, L1 stage worms on nonstarved plates were washed off, filtered through a 10 µm filter (pluriSelect, pluriStrainer 10 µm), collected via centrifugation, transferred to seeded plates, and grown to the desired stage.

### RNAi interference

*Escherichia coli* HT115(DE3) strains from a previously developed library (Geneservices, United Kingdom), containing the RNAi constructs of interest, were utilized for the RNAi experiments (Timmons *et al.* 2001). The same parent strain [HT115(DE3)] containing the empty vector L4440 was used as a control. RNAi experiments were conducted by feeding worms from the L1 to L4 stage at 16°C on agar plates with bacteria expressing the double-stranded RNA corresponding to the gene to be knocked down. Generally, RNAi bacteria were cultured to stationary phase, followed by the addition of 1 mM isopropyl β-D-1-thiogalactopyranoside (IPTG), and then the culture was incubated for another hour. Subsequently, the culture was concentrated 5-fold before being seeded onto NG agar plates supplemented with 100 µg/mL carbenicillin and 1 mM IPTG for 5 h to induce dsRNA expression at 37°C. Double RNAi knockdowns were obtained by mixing the 2 bacterial RNAi cultures in a 1:1 ratio.

### Infection with bacteria

Synchronized populations of worms, in the L4 larval or young adult stage, previously exposed to RNAi bacteria, were exposed to *E. faecalis* strain OG1RF for 16–20 h on brain heart infusion (BHI) agar plates or *P. aeruginosa* strain PA14 for 7 h on NG agar plates. *Escherichia coli*HT115, on NG agar plates, was employed as the nonpathogen control. The incubation temperature was 25°C, and all experiments were independently performed 3 times.

### Screen for genes that affect SKN-1 activity on pathogen

The *Pgcs-1::gfp* strain was screened on *P. aeruginosa* whereas the *Pgst-4::gfp* strain was screened on *E. faecalis* following RNAi knockdown. To carry out the screen, L1 animals were harvested and dispensed into liquid NGM medium containing the bacterial food expressing the RNAi clone of interest (see below for how initial RNAi clones were chosen). At the L4 stage, the animals were moved onto agar plates containing *E. faecalis* or *P. aeruginosa*. Following a 6-h (*P. aeruginosa*) or 20-h (*E. faecalis*) exposure, the animals were washed and put into 96-well plates. Animals were paralyzed by the addition of 50 mM tetramisole and examined using a dissecting fluorescent microscope. Positive (control RNAi) and negative (*skn-1* RNAi) controls were included in every batch of RNAi clones screened. RNAi clones that caused a visible decrease in the high level of intestinal reporter fluorescence normally induced by pathogen exposure were confirmed by performing two more biological replicates. For these replicates, a BioTek Cytation 5 imaging plate reader was used as described below.

The RNAi clones to screen were chosen based on the following criteria. First, WormMine (http://intermine.wormbase.org/tools/wormmine) was used to identify 3,135 genes with evidence of intestinal expression for the following reasons. (1) We were looking at SKN-1 activation following pathogen infection of this organ, and therefore, we reasoned it is likely that relevant regulators of SKN-1 are also in the intestine. (2) RNAi of intestinal genes works well, unlike RNAi of genes expressed in tissues such as the neurons. The list was further narrowed to genes that were annotated with keywords related to signaling components and factors that could be involved in redox and cysteine metabolism (1,077). Finally, the list was whittled to include only the genes available as clones in the *C. elegans* RNAi library (933) (Fraser *et al.* 2000). Eighteen genes, with no prior evidence of involvement in SKN-1 regulation and not compromised by severe growth and developmental defects, were identified including *cdc-48.1*, *cdc-48.2*, and *nipi-3* (Wu *et al.* 2021).

### Fluorescence quantification (confocal microscopy and BioTek)

To assess the expression of *Pgst-4::GFP* and *Pgcs-1::GFP*, SKN-1-B/C::GFP, SKN-1-A::GFP, *Prpt-3::GFP*, and *Phsp-4::GFP*, an Olympus FLUOVIEW FV 3000 confocal microscope imaging system, equipped with Fluoview FV315-SW software, was utilized. Animals were exposed to *E. faecalis* or *P. aeruginosa* at 25°C for 16 or 7 h, respectively, with *E. coli* exposure serving as the control condition. Following paralysis with 25 mM levamisole, anesthetized worms were mounted on 2% agarose pads for microscopy visualization. The mean level of GFP fluorescence intensity per animal was quantified using a Cytation 5 imaging plate reader (BioTek). Briefly, approximately 50−80 worms in 100 µL M9 buffer with 25 mM levamisole were transferred to a 96-well plate (Corning Incorporated Costar, 3603). Quantification of the GFP signal was performed utilizing Gen5 3.08 software, and 3 biological replicates were used for each experiment.

SKN-1B/C::GFP and SKN-1-A::GFP expression was analyzed by the abovementioned fluorescent microscopy of worms exposed to *E. faecalis* for 16 h and *P. aeruginosa* for 7 h. To discriminate intestinal autofluorescence from SKN-1B/C::GFP and SKN-1A::GFP fluorescence, we used a combination of the EGFP and mCherry filter sets. This combination allowed autofluorescence to be detected as a yellow to red signal. The degree of SKN-1B/C::GFP nuclear localization in the intestinal cells was scored as previously described (An and Blackwell 2003; Inoue *et al.* 2005). Briefly, no nuclear localization in the anterior or posterior of the worm, and nuclear localization in all intestinal cells are categorically indicated by low, medium, and high, respectively. All fluorescence microscopy experiments shown were independently repeated at least 3 times.

### Killing assays

Killing assays were conducted as previously described (Tan *et al.* 1999; Garsin *et al.* 2001). Briefly, *E. faecalis*OG1RF grown in BHI to late log phase was seeded on BHI agar plates with 10 µg/mL gentamycin and 10 µg/mL nystatin and incubated at 37°C for 24 h. For *P. aeruginosa* killing assays, *P. aeruginosa*PA14 was cultured in Luria broth (LB), seeded on slow-killing plates, and incubated, first, for 24 h at 37°C and then for 14 h at 25°C. The killing assay plates contained a ring of palmitic acid (10 µg/mL in ethanol), which precipitates out of solution and forms a physical barrier that prevented the worms from escaping. A total of 90–180 L4 larvae were transferred to 3 replica plates. Worms were scored as live and dead at 24-h intervals starting at day 3.

### RNA isolation and qRT-PCR analysis

RNA was extracted from about 1,000 L4 animals exposed to *E. faecalis*OG1RF for 16 h or *P. aeruginosa*PA14 for 8 h. RNA from animals exposed to *E. coli*HT115 was extracted as a control at the same time points. The RNA was extracted with Direct-zol RNA Miniprep plus (Zymo Research), and cDNA synthesis was executed using Primer Script RT Master Mix (TakaRa cat# RR036A). qRT-PCR was performed on an CFX 96 Real-Time system (Bio-Rad) using SYBR Green Master mix. The primer sequences are listed in Supplementary Table 1. All values were normalized to *act-1*

### Semiquantitative RT-PCR

Following RNA extraction and cDNA synthesis as described above, a conventional PCR was conducted to quantify the spliced and nonspliced forms of XBP-1. For amplification, 10 ng/μL of cDNA was mixed with Platinum SuperFi II DNA Polymerase High Fidelity PCR Enzyme (Thermo Fisher), following the manufacturer's specifications. The primers used were forward 5′-TGCATCTACCAGAACGTCGT and reverse 5′-CGGAGTTGGTTGCTGATGTT. The thermal cycles for amplification included an initial step at 98°C for 30 s, followed by 30 cycles of 98°C for 10 s, 60°C for 10 s, 72°C for 15 s, and a final extension cycle at 72°C for 5 min. Visualization and quantification of the bands of interest (xbp-1U 176bp, xbp-1S 153bp) were performed using a 2.5% agarose gel. ImageJ software was utilized for quantification.

### Western blot analysis

Worms of each genotype (2,000–3,000 L4 stage individuals post pathogen exposure) were collected and washed with M9 buffer, followed by boiling in a homemade sample buffer and centrifugation. The resulting supernatants were flash-frozen in dry ice and then heated at 80°C for 10 min. Proteins were resolved on a 12% SDS-polyacrylamide gel by electrophoresis, transferred to a PVDF membrane, and incubated with rabbit anti-phospho-p38 MAPK (Cell Signaling, #9211) at a 1:2,000 dilution or mouse anti-tubulin (Sigma, #T9026) at a 1:4,000 dilution. Following washing, the blots were incubated with 1:2,000 secondary HRP-conjugated anti-mouse (for anti-tubulin) or anti-rabbit (for anti-phospho-p38) antibody for 1 h and subsequently washed 3 times for 3-min intervals. Blots were developed using SuperSignal West Atto Ultimate Sensitivity Substrate (Thermo Fisher, A38555) and visualized using a ChemiDoc MP Imaging System (Bio-Rad). Image lab (software) was used to quantify the intensity of the immunoblot bands.

### Statistical analysis

GraphPad Prism 10 was used for data analysis. Following categorical scoring of confocal images, statistical significance was determined by *χ*^2^ of Fischer's exact tests. For quantification of fluorescent measures, qRT-PCR/RT-PCR and western blot significance were determined using unpaired *t* tests for experiments with only 2 samples or 2-way ANOVA followed by Tukey's multiple comparison tests for experiments with multiple samples. Mantel–Cox log rank analysis was used to compare survival curves and to calculate the median survival. Comparisons of interest and their statistical significance are indicated in the figures. For all statistical tests, *P* < 0.05 was considered statistically significant (*P < 0.05, **P < 0.01, ***P < 0.001, ****P < 0.0001).

## Results

### CDC-48 is required for activation of SKN-1 genes following pathogen exposure

Both *P. aeruginosa* and *E. faecalis* colonize the intestine of *C. elegans* and induce SKN-1 regulated genes (Hoeven *et al.* 2011; Papp *et al.* 2012). As described in previous work, the *Pgst-4::GFP* reporter is more strongly induced by *E. faecalis* whereas the *Pgcs-1::GFP* is more strongly induced by *P. aeruginosa* (Hoeven *et al.* 2011). Genes that affect SKN-1 activation following exposure of these reporter strains to these pathogens were identified in a screen (*Materials and methods* and Wu *et al.* 2021). Specifically, we screened 933 genes with evidence of intestinal expression and connections to signaling components and factors that could be involved in redox and cysteine metabolism (see *Materials and methods* for details). We observed that RNAi of *cdc-48* caused loss of SKN-1 activity. Specifically, animals were exposed to a *cdc-48* RNAi construct predicted to target both *cdc-48.1* and *cdc-48.2* (Zou *et al.* 2014). These 2 homologs are 88% identical and likely arose from a gene duplication event.

**Fig. 1.**
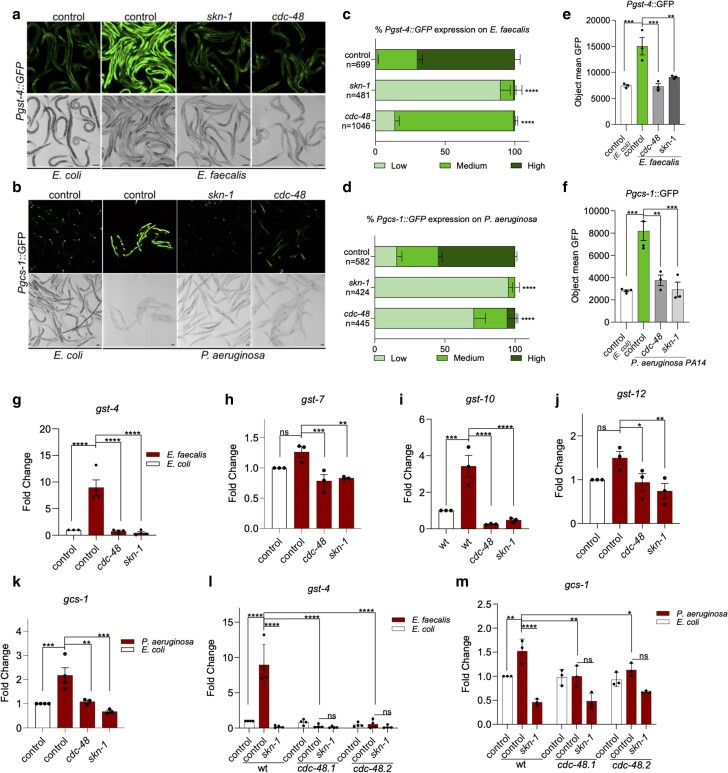
CDC-48 is required for activation of SKN-1 following pathogen exposure. a and b) *Pgst-4::GFP* and *Pgcs-1::GFP* expression patterns in worms exposed to *E. faecalis*, *P. aeruginosa*, or *E. coli* following RNAi knockdown of the indicated genes. Scale bars are 100 µm. c and d) The degree of *Pgst-4::GFP* and *Pgcs-1::GFP* expression was scored based on GFP intensity levels (low, medium, high), and the percentage of worms in each category was calculated. The number of worms quantified and used in scoring is indicated by (*n*). The asterisks indicate the statistical significance of the levels of *Pgst-4::GFP* and *Pgcs-1::GFP* observed in *cdc-48* and *skn-1* RNAi animals as compared to control RNAi animals. e and f) Quantification of GFP fluorescence in animals with *Pgst-4::GFP* and *Pgcs-1::GFP* expression reporters, as shown in a) and b), was performed using a Cytation 5 imaging plate reader. The *y*-axis represents the average pixel count measured in nematodes following pathogen exposure. Analysis was conducted on 3 biological replicates, each with at least 50 worms. Error bars indicate the standard error of the mean (SEM). The asterisks indicate the statistical significance of the bracketed comparisons. g–k) qRT-PCR quantification analysis of SKN-1 regulated genes in wild-type N2 worms following control, *cdc-48* and *skn-1* RNAi, and infection with *E. faecalis* or *P. aeruginosa*. Gene expression values were normalized to the *act-1* housekeeping gene and are relative to the control RNAi animals exposed to *E. coli* and set to 1. Error bars represent the SEM of the biological replicates. The asterisks indicate the statistical significance of the bracketed comparisons. l and m) *gst-4* and *gcs-1* expression levels were measured by qRT-PCR in N2 wild-type, *cdc-48.1(tm544)*, and *cdc-48.2(tm659)* mutant worms exposed to pathogens following control or *skn-1* RNAi. Gene expression data were processed as described above, and the asterisks indicate the statistical significance of the bracketed comparisons. *P < 0.05; **P < 0.01; ***P < 0.001; ****P < 0.0001; ns, not significant.

Representative pictures of *Pgst-4::GFP* animals exposed to control, *skn-1*, and *cdc-48* RNAi gene followed by exposure to *E. faecalis* are shown in Fig. 1a. Included as an additional control are animals on *E. coli*, not exposed to pathogen. Both categorical scoring (Fig. 1c) and fluorescent signal measurements with an imaging plate reader (Fig. 1e) were used to quantify the signal. Exposure to *E. faecalis* induced the *Pgst-4::GFP* reporter as observed previously, but RNAi of *cdc-48* significantly prevented this induction as did *skn-1* RNAi, which served as a positive control for these experiments. The results with *Pgcs-1::GFP* animals exposed to *P. aeruginosa* were similar. Exposure to *P. aeruginosa* increased expression of this reporter, but previous exposure to *cdc-48* RNAi prevented this induction (Fig. 1b, d, and f). While as previously observed (Hoeven *et al.* 2011), the induction of *Pgcs-1::GFP* was not as strong on *E. faecalis*, a loss of induction following *cdc-48* RNAi was also discernable (Supplementary Fig. 1).

In addition to using SKN-1 regulated reporters, expression of endogenous SKN-1 regulated genes on pathogen was examined by qRT-PCR (Hoeven *et al.* 2011). As shown in Fig. 1g–k, the induction of *gst-4*, *gst-7*, *gst-10*, *gst-12*, and *gcs-1* following pathogen exposure was significantly reduced by *cdc-48* RNAi like the positive control, *skn-1* RNAi.

To determine if the effects on SKN-1 gene activation were specific to one of the *cdc-48* genes, we obtained animals containing the deletion alleles, *cdc-48.1(tm544)* and *cdc-48.2(tm659)* (Zou *et al.* 2014), and measured *gst-4* and *gcs-1* gene expression following exposure to *E. faecalis* and *P. aeruginosa*, respectively. Note that it is not possible to cross these strains and obtain a double deletion because CDC-48 is essential (Zou *et al.* 2014). However, in both single deletion mutants, we observed that *gst-4* and *gcs-1* failed to be significantly induced when the strains were exposed to pathogen (Fig. 1l and m). The results indicate that the presence of both CDC-48.1 and CDC-48.2 is required for SKN-1 gene activation; the presence of just one cannot compensate for the loss of the other.

### CDC-48 is required for SKN-1 mediated pathogen resistance

**Fig. 2.**
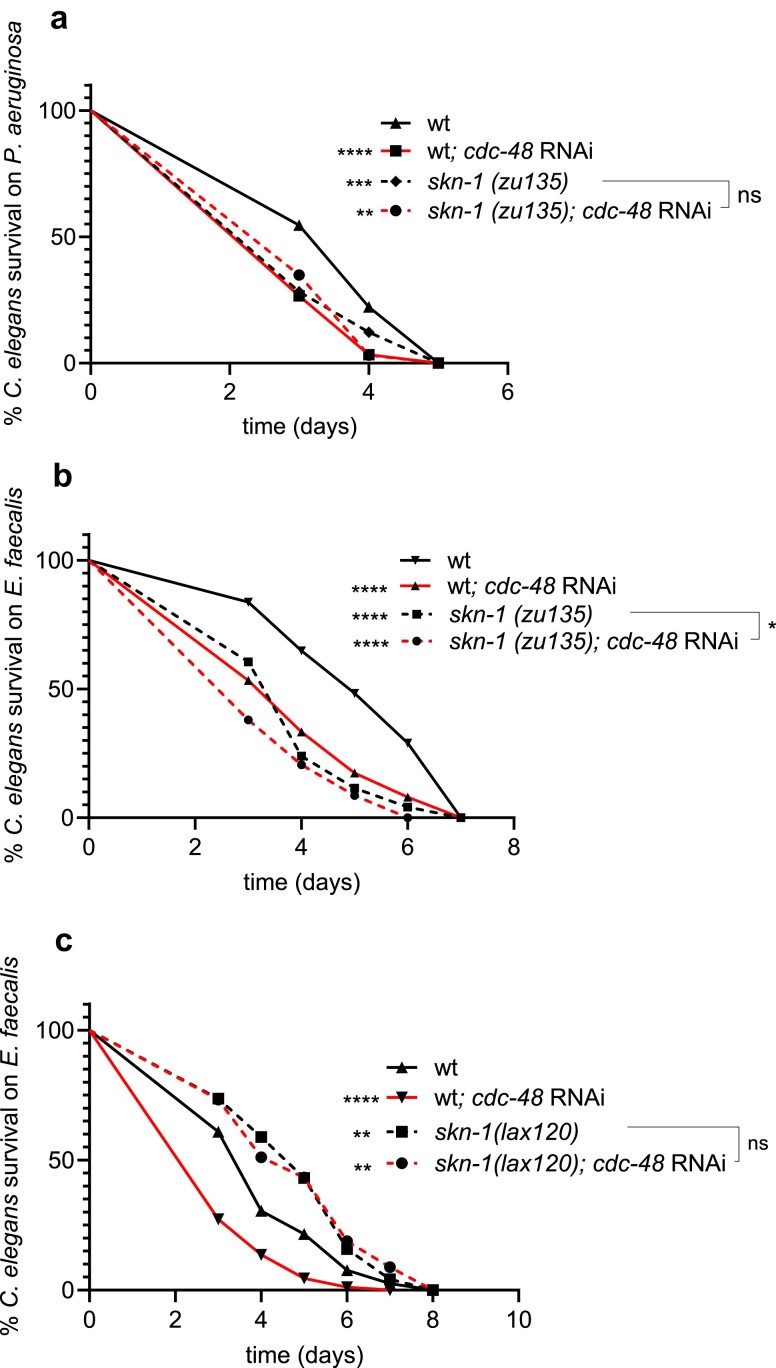
CDC-48 is required for SKN-1 mediated pathogen resistance. a) Survival curves of wild-type and *skn-1(zu135)* worms exposed to control or *cdc-48* RNAi followed by infection with *P. aeruginosa* a) or *E. faecalis* b). c) Survival curves of wild-type and *skn-1(lax120)* worms on *E. faecalis*. Note that in all the survival assays, the worms were additionally exposed to *cdc-25.1* RNAi to sterilize them (Hoeven *et al.* 2011). The data shown in each panel are representative of 2 or 3 independent trials. Sample sizes, median survival, and *P*-values of all trials are given in Supplementary Table 2.

CDC-48 and SKN-1 were previously both shown to be required for optimal resistance to pathogen (Hoeven *et al.* 2011; Zou *et al.* 2014). To explore whether CDC-48 and SKN-1 associated pathogen susceptibility phenotypes were consistent with these factors operating in the same pathway, genetic epistasis was performed using a loss-of-function allele of *skn-1*. Knockdown of *cdc-48* in this background did not significantly increase the sensitivity of the *skn-1* mutant to *P. aeruginosa* (Fig. 2a) and only slightly increased sensitivity to *E. faecalis* (Fig. 2b). We additionally tested a *C. elegans* strain containing a gain-of-function allele, *skn-1(lax120)* (Paek *et al.* 2012), that has increased resistance to pathogen (Wu *et al.* 2021). Suppression of *cdc-48* by RNAi in *skn-1(lax120)* animals did not affect the increased resistance to *E. faecalis* exhibited by this strain (Fig. 2c). Altogether, the data are consistent with a model in which CDC-48 is required for SKN-1 mediated pathogen resistance.

### SKN-1C, but not SKN-1a, nuclear translocation, and activation is triggered by pathogen exposure and affected by loss of CDC-48

**Fig. 3.**
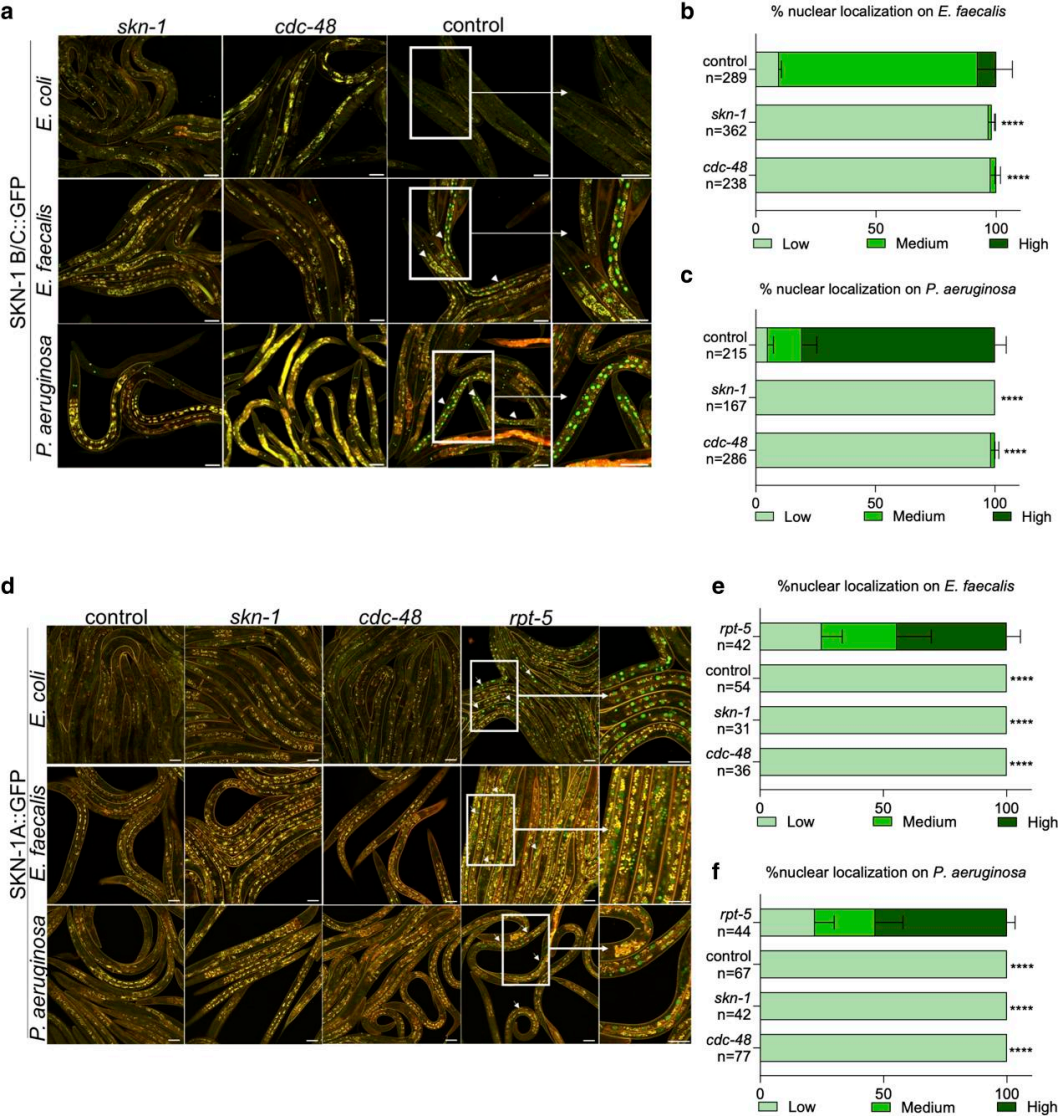
SKN-1C, but not SKN-1A, nuclear localization is triggered by exposure to pathogen and affected by loss of CDC-48. a) Worms containing the integrated SKN-1B/C::GFP transgene were exposed to *E. faecalis* or *P. aeruginosa*. SKN-1B/C::GFP localization was observed by fluorescence microscopy. Example intestinal nuclei displaying nuclear localization are marked by white arrows and white boxes mark magnified insets. Scale bars are 100 μm. b and c) SKN-1C nuclear localization was scored based on the GFP signal (low, medium, high), and the percentage for each category was quantified. The number of worms used in scoring each experimental condition is indicated (*n*). Levels of SKN-1B/C::GFP nuclear localization in the intestines of animals on pathogen following *cdc-48* and *skn-1* RNAi were compared to control RNAi. ****P < 0.0001. d) Worms containing the integrated SKN-1A::GFP transgene were exposed to *E. faecalis* or *P. aeruginosa*. SKN-1A::GFP localization was observed by fluorescence microscopy, with nuclear localization only visible in the positive control (*rpt-5* RNAi), as marked with white arrows. White boxes mark magnified insets. Scale bars are 100 μm. e and f) SKN-1A nuclear localization was scored based on the GFP signal (low, medium, high), and the percentage for each category was quantified. The number of worms used in scoring each experimental condition is indicated (*n*). Levels of SKN-1A::GFP nuclear localization in the intestines of animals on pathogen following *cdc-48* and *skn-1* RNAi were compared to control RNAi. ****P < 0.0001.

To investigate the effects of the SKN-1 isoforms and CDC-48 on SKN-1 activation, we used GFP translational fusions to different isoforms of SKN-1 and observed their nuclear translocation. SKN-1 nuclear translocation into the intestinal nuclei upon pathogen exposure was previously observed using a *C. elegans* strain containing a translational fusion to 2 of the expressed isoforms, SKN-1B/C::GFP (An and Blackwell 2003). In this strain, constitutive nuclear localization of SKN-1B/C::GFP is observed in the ASI neurons associated with the SKN-1B isoform, but inducible intestinal nuclear localization of the SKN-1C isoform can be observed. As shown in Fig. 3a, exposure to *E. faecalis* and *P. aeruginosa* induced nuclear localization of the SKN-1B/C::GFP transgene in the intestinal cells, consistent with previous work (Hoeven *et al.* 2011; McCallum *et al.* 2016; Wu *et al.* 2021). To test if loss of CDC-48 prevented this translocation, this strain was exposed to *cdc-48* RNAi prior to exposure to *E. faecalis* or *P. aeruginosa* and the animals observed by confocal microscopy (Fig. 3a). Loss of CDC-48 was associated with a significant inhibition of nuclear translocation that was quantified by categorical scoring in comparison to control RNAi animals (Fig. 3b and c).

In a previous study, a SKN-1A::GFP transgene was introduced into the genome driven by a constitutive promoter (*Prpl-28*::SKN-1A::GFP). Loss of proteosome homeostasis, by RNAi of a proteosome subunit-encoding gene (*rpt-5*) or by addition of the chemical bortezomib, drove nuclear localization of SKN-1A::GFP and was visualized occurring in the intestinal as well as the hypodermal tissue (Lehrbach and Ruvkun 2016). To test if exposure to *E. faecalis* and *P. aeruginosa* caused nuclear translocation SKN-1A, we obtained this strain and exposed the animals to *E. faecalis* or *P. aeruginosa* (Fig. 3d). Pathogen exposure did not induce any visible localization of SKN-1A::GFP. As a positive control, animals were exposed to *rpt-5* RNAi. As previously reported (Lehrbach and Ruvkun 2016), nuclear localization of SKN-1A::GFP was visible following *rpt-5* RNAi and was not further affected by subsequent pathogen exposure (Fig. 3b). Additionally, a reporter strain for SKN-1A activity was investigated in which the promoter of *rpt-3*, a gene encoding a proteosome subunit, was fused to GFP (Lehrbach and Ruvkun 2016). As shown in Supplementary Fig. 2, exposure to neither *E. faecalis* nor *P. aeruginosa* induced expression of this SKN-1A reporter. However, expression was clearly visible in animals exposed to *rpt-5* RNAi, the positive control, both by confocal microscopy (Supplementary Fig. 2a) and by quantifying the signal with an imaging plate reader (Supplementary Fig. 2b and c). In total, these data suggest that exposure to these pathogens does not activate SKN-1A. However, consistent with previous studies, exposure to these pathogens induces SKN-1C nuclear localization. Moreover, loss of CDC-48 prevents this nuclear localization strongly suggesting that CDC-48's negative effects on SKN-1 activity occur mainly via the SKN-1C isoform under these conditions.

### Loss of *cdc-48* activates the UPR associated with ER stress

Because CDC-48 and ERAD play a critical role in removing misfolded proteins from the ER, it is likely that targeting CDC-48 by RNAi or by genetic mutation causes ER stress and activation of the unfolded protein response (UPR^ER^). Interestingly, previous work reported that activation of the UPR^ER^ inhibited SKN-1's protective response to oxidative stress and vice versa (Hourihan *et al.* 2016). Based on this knowledge, we hypothesized that loss of CDC-48 was activating the UPR^ER^, thereby indirectly suppressing SKN-1 activation on pathogen.

**Fig. 4.**
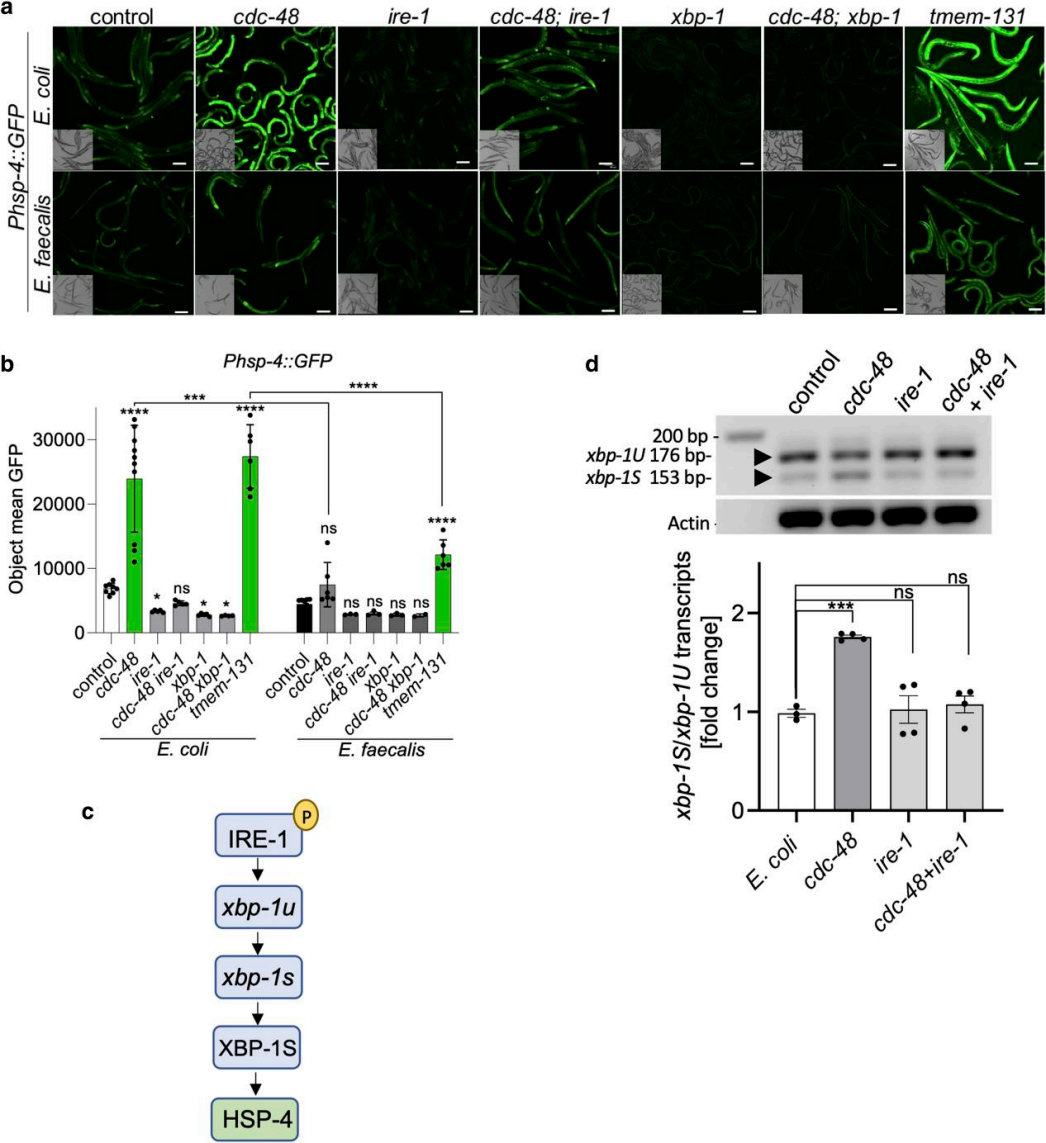
Loss of CDC-48 activates the UPR associated with ER stress. a) Confocal microscopy of worms containing the *Phsp-4::GFP* transgene (ER stress reporter) following RNAi of the indicated genes and exposure to *E. coli* or *E. faecalis*. Scale bars are 100 µm. b) Quantification of the mean GFP fluorescence for animals in a) with a Cytation 5 imaging plate reader. The *y*-axis represents the mean pixel count measured in the worms. Analysis was conducted on 3 biological replicates, each with at least 50 worms. Error bars indicate the standard error of the mean (SEM). For each bacterial exposure, the statistical significance of the RNAi condition compared to the control is denoted above the bars. Additional comparisons are indicated by brackets: *P < 0.05; ***P < 0.001; ****P < 0.0001; ns, not significant. c) Model for the regulation of HSP-4 by the UPR^ER^ pathway as established in previous work. d) Semiquantitative PCR analysis of *xbp-1S/xbp-1U*. A reverse transcription PCR was performed on animals following *cdc-48*, *ire-1*, or *cdc-48; ire-1* RNAi to measure the transcript levels of *xbp-1*. The band intensities were densitometrically analyzed using ImageJ software, and, following normalization with housekeeping transcript actin 1, the levels of *xbp-1S* relative to *xbp-1U* were calculated with the control RNAi condition set to 1. Error bars represent the SEM of the averaged biological replicates. The asterisks indicate the statistical significance of the bracketed comparisons. ***P < 0.001; ns, not significant.

To test this hypothesis, animals containing the *Phsp-4::GFP* reporter were exposed to *cdc-48* RNAi. Activation of this reporter was apparent, both by confocal microscopy (Fig. 4a) and when the fluorescent signal was measured using an imaging plate reader (Fig. 4b). Activation was also observed following knockdown of *tmem-131*, which was previously reported to activate the ER stress response and was utilized as a positive control (Zhang *et al.* 2020). Additionally, upregulation of the endogenous *hsp-4* gene, as well as 2 other genes reported to be upregulated by this pathway, *hsp-3* and *ckb-2* (Kapulkin *et al.* 2005; Caruso *et al.* 2008; Morrison *et al.* 2014), was observed by qRT-PCR following *cdc-48* RNAi (Supplementary Fig. 3).

Interestingly, like *cdc-48* RNAi, RNAi of *tmem-131* in animals containing the *Pgst-4::GFP* reporter reduced activation on pathogen (Supplementary Fig. 4). The observation is consistent with the idea that inducers of ER stress inhibit the oxidative stress response. Also consistent with the model of mutual inhibition was the observation that activation of *Phsp-4::GFP* by *cdc-48* RNAi (and *tmem-131* RNAi) was reduced following pathogen (*E. faecalis*) exposure compared to animals exposed to *E. coli* (Fig. 4b). As exposure to pathogens like *E. faecalis* causes ROS production, generates oxidative stress, and activates SKN-1, we postulated that these experimental conditions inhibit the UPR^ER^ induction caused by *cdc-48* RNAi in a manner analogous to what was previously observed using chemical mediators of oxidative and ER stress (Hourihan *et al.* 2016).

As diagramed in Fig. 4c, HSP-4 activation is driven by a pathway consisting of the ER transmembrane kinase/RNase IRE-1. The presence of unfolded proteins in the ER drives the oligomerization and autophosphorylation of IRE-1 resulting in the activation of its RNase domain (Ron and Walter 2007; Wang and Kaufman 2014). The mRNA encoding XBP-1 is spliced by IRE-1, generating the active form of this transcription factor that drives the expression of genes related to the UPR^ER^, including *hsp-4* (Taylor and Dillin 2013). The expression of *Phsp-4::GFP* resulting from RNAi of *cdc-48* was dependent on IRE-1 and XBP-1 as predicted by this pathway; simultaneous RNAi of *cdc-48* and *ire-1* or *cdc-48* and *xbp-1* reduced expression of *Phsp-4::GFP* (Fig. 4a and b). To examine the effects of *cdc-48* RNAi on IRE-1-driven XBP-1 splicing more directly, the levels of spliced and unspliced XBP-1 mRNA were probed. As expected, levels of the spliced form of the mRNA increased following *cdc-48* RNAi in a manner dependent on IRE-1. Simultaneous RNAi against *cdc-48* and *ire-1* abrogated the effect (Fig. 4c).

### Loss of CDC-48 reduces signaling through the IRE-1/p38 MAPK pathway

**Fig. 5.**
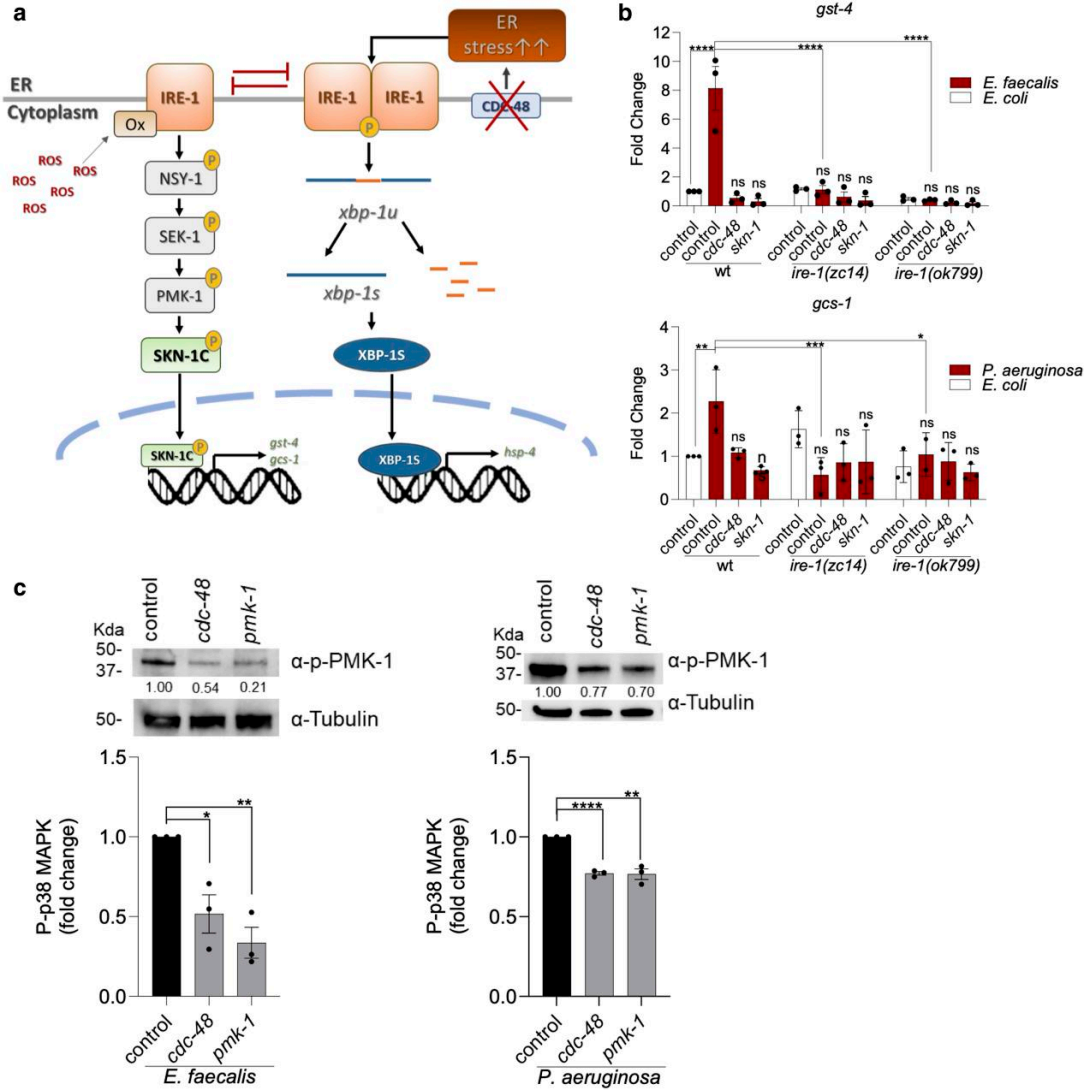
Loss of CDC-48 reduces signaling through the IRE-1/p38 MAPK pathway. a) Model for how loss of CDC-48, by inducing the UPR^ER^, negatively affects the p38 MAPK pathway and SKN-1C activation. See main text for details. b) qRT-PCR quantification of *gst-4* and *gcs-1* transcripts in wild-type N2, *ire-1(zc14)*, and *ire-1(ok799)* animals following control, *cdc-48*, and *skn-1* RNAi and exposure to *E. faecalis* or *P. aeruginosa*. Gene expression values were normalized to the *act-1* housekeeping gene and are relative to the wild-type control RNAi animals on *E. coli* set to 1. Error bars represent the SEM of the biological replicates. The asterisks indicate the statistical significance of the bracketed comparisons. c) Immunoblot analysis of PMK-1 phosphorylation levels using an α-phospho-p38 antibody and an α-tubulin antibody (loading control) from lysates of the indicated animals exposed to *E. faecalis* or *P. aeruginosa*. Shown are representative blots. The average of the band intensities relative to α-tubulin from 3 biological replicates are presented below. The asterisks indicate the statistical significance of the bracketed comparisons. *P < 0.05; **P < 0.01; ***P < 0.001; ****P < 0.0001; ns, not significant.

As mentioned, activation of the UPR^ER^ was shown to inhibit SKN-1 activation and vice versa (Hourihan *et al.* 2016). The model for how mutual inhibition occurs, depicted in Fig. 5a, centers around the crucial signaling role IRE-1 plays in both processes. In addition to driving the UPR^ER^ response, as described above, IRE-1 was shown to drive the p38 MAPK pathway that promotes SKN-1 activity in a mutually exclusive manner. These experiments were done using sodium arsenite (AS) as the mediator of oxidative stress and tunicamycin (TM) to induce ER stress. The increase in intracellular ROS caused by AS exposure activated IRE-1 sulfenylation, promoting antioxidant function and inhibiting UPR function (Hourihan *et al.* 2016). We postulated that the same mechanism is in effect following loss of CDC-48 (ER stress) and exposure to pathogen (oxidant stress). To investigate, we examined the expression of SKN-1 regulated genes in 2 *ire-1* mutant strains of *C. elegans* following pathogen exposure. As observed in Fig. 5b, expression of *gst-4* and *gcs-1* was not significantly induced in both *ire-1* mutant backgrounds following exposure to *E. faecalis* and *P. aeruginosa*, respectively, indicating that IRE-1 is required.

The p38 MAPK pathway was a previously established requirement for SKN-1 activity on the tested pathogens (Hoeven *et al.* 2011), and IRE-1 was shown to mediate its activating effects on SKN-1 via p38 MAPK signaling (Hourihan *et al.* 2016) (Fig. 5a). Therefore, if loss of *cdc-48* is inhibiting SKN-1 activity on pathogen by the mutual inhibition model, the activation of the UPR^ER^ by loss of CDC-48 is predicted to reduce signaling through the p38 MAPK cascade. As shown in Fig. 5c, *cdc-48* RNAi significantly reduced the levels of phosphorylated PMK-1, the p38 MAPK, following exposure to *E. faecalis* and *P. aeruginosa*. As a control, RNAi against *pmk-1* also reduced the amount of phosphorylated PMK-1 as expected. In total, the data support the model depicted in Fig. 5a in which loss of *cdc-48* causes ER stress and the recruitment of IRE-1 to the UPR^ER^ signaling pathway, thereby reducing the amount available to promote SKN-1 activation through the p38 MAPK signaling cascade.

## Discussion

In this work, we established that activation of the SKN-1 genes associated with oxidative stress requires the presence of CDC-48 and loss of CDC-48 renders the animals more sensitive to pathogen. In a previous study examining the function of autophagy during infection, loss of CDC-48 was also shown to increase sensitivity to *P. aeruginosa* (Zou *et al.* 2014). Moreover, work focused on cofactors that are part of the of the ERAD complex, NPL-4 and URD-1, also reported susceptibility to *P. aeruginosa* (Rao *et al.* 2023). Our data herein suggest that the CDC-48 dependent pathogen sensitivity phenotype is due, in part, to loss of SKN-1 activation under these conditions. Furthermore, by using translational fusions to different SKN-1 isoforms and examining their relocalization following pathogen exposure, we learned that SKN-1C is the likely mediator of these effects. Nuclear translocation of a SKN-1B/C::GFP translational fusion in the intestine was observed in response to pathogen and shown to be dependent on CDC-48. However, nuclear translocation of a SKN-1A::GFP reporter failed to be stimulated by these conditions. Additionally, a reporter for SKN-1A transcriptional activity was not activated. Thus, pathogen exposure, at least exposure to *E. faecalis* and *P. aeruginosa*, appears to activate the SKN-1C, but not the SKN-1A isoform in the intestine.

When considering the mammalian orthologs of the SKN-1 isoforms, it is becoming increasingly recognized that SKN-1C is an ortholog of Nrf2. Shared features include localization primarily to the cytoplasm and induction of the antioxidant and xenobiotic defense responses associated with this family of transcription factors. In contrast, SKN-1A is most similar to the Nrf1 ortholog in that it is localized to the ER and maintains protein homeostasis. SKN-1A was shown to be specifically activated by disrupting proteosome function using the chemical bortezomib or by reducing the expression of proteosome subunits (Lehrbach and Ruvkun 2016). More recently, oleic acid (OA) was found to be another inducer of SKN-1A activation in a mechanism independent of proteosome activity and attributed to OA enhancing ERAD activity via lipid droplets (Castillo-Quan *et al.* 2023). Further investigations into the conditions that induce SKN-1, and specifically isoforms of SKN-1, will be of interest and have potential impact on the understanding the mammalian Nrf orthologs.

The mechanism behind the mutually inhibitory oxidative stress response and UPR following exposure to chemical inducers was shown to center on the IRE-1 kinase (Fig. 5a) (Hourihan *et al.* 2016). It is well established that unfolded proteins induce the UPR by causing the oligomerization and autophosphorylation of IRE-1, activating its RNase activity and leading to the processing of the XBP-1 transcript. However, in elegant and novel work, modification of IRE-1 by sulfenylation was discovered to be necessary to drive activation of the p38 MAPK pathway as an alternative response (Hourihan *et al.* 2016). In addition to IRE-1 sulfenylation, activation also required sulfenylation of NSY-1, which is thought to occur by interaction with IRE-1, via TRF-1, resulting in proximity to an oxidizing environment provided by the ER, closely associated mitochondria, and/or activated BLI-3 (Hourihan *et al.* 2016). Our data in this work and in previous studies align with a similar mechanism activating the oxidative stress response following exposure to *E. faecalis*, *P. aeruginosa*, and the human fungal pathogen *Candida albicans* (Hoeven *et al.* 2011; van der Hoeven *et al.* 2015). Specifically, a requirement for IRE-1 to activate SKN-1 regulated genes following pathogen exposure was observed (Fig. 5b). In contrast, infection with the pathogen *Streptococcus mitis* was found to activate SKN-1 independently of IRE-1 (Naji *et al.* 2018). A likely explanation is that *S. mitis*, unlike *E. faecalis* and *P. aeruginosa*, produces large amounts of hydrogen peroxide (Naji *et al.* 2018), and this membrane-diffusible ROS might result in sulfenylation of NSY-1 independent of the need for IRE-1 to localize NSY-1 to an oxidizing subcellular environment.

Overall, this work contributes to the growing body of literature revealing the importance of maintaining proteostasis during pathogen infection, a process to which ERAD function contributes. Moreover, the data support a model in which changes in ERAD function, caused in our study by loss of CDC-48, affect immune response in part by influencing SKN-1 activation. In conclusion, the need to correctly activate and coordinate stress response pathways during infectious conditions is crucial in order to maintain organismal health and homeostasis.

## Supplementary Material

iyae131_Supplementary_Data

## Data Availability

All data necessary for confirming the conclusions of this article are represented within the article. The raw data that underlie the figures are provided in Supplementary Table 2. The sources for all strains and reagents are provided in Supplementary Table 1. Supplemental material available at GENETICS online.
