# Supplementary material for: SKN-1 activation during infection of Caenorhabditis elegans requires CDC-48 and endoplasmic reticulum proteostasis
Source: Genetics. 2024 Aug 21;228(3):iyae131. doi: 10.1093/genetics/iyae131 (PMC11538416; doi:10.1093/genetics/iyae131)
Supplement: iyae131_Supplementary_Data [file iyae131_supplementary_data.zip › Supplemental_Figures_GENETICS-2024-307217.pdf]

A

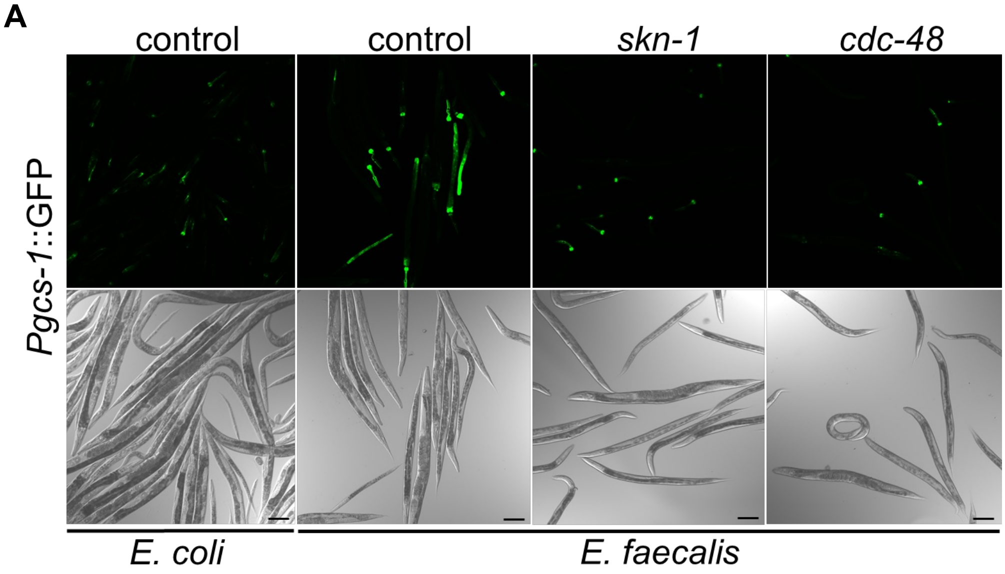

B

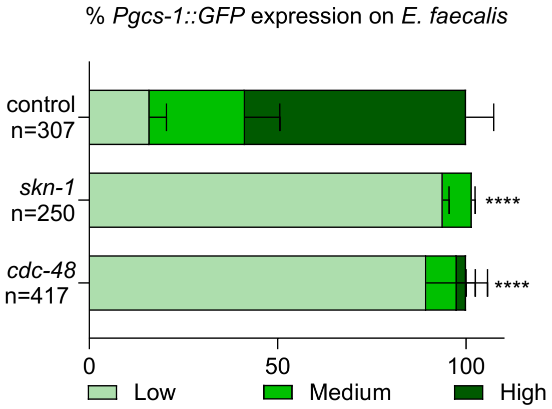

Figure S1. CDC-48 is required for activation of SKN-1 following pathogen exposure
(A) *Pgcs-1::GFP* expression pattern in worms exposed to *E. faecalis* and *E. coli*. Scale bars are 100 μm. (B) The degree of *Pgcs-1::GFP* expression was scored based on GFP intensity levels (Low, Medium, High), and the percentage of worms in each category was calculated. The number of worms quantified and used in scoring is indicated by (n). The asterisks indicate the statistical significance of the levels of *Pgcs-1::GFP* fluorescence observed in *cdc-48* and *skn-1* RNAi animals compared to control RNAi animals. ****$p < 0.0001$.

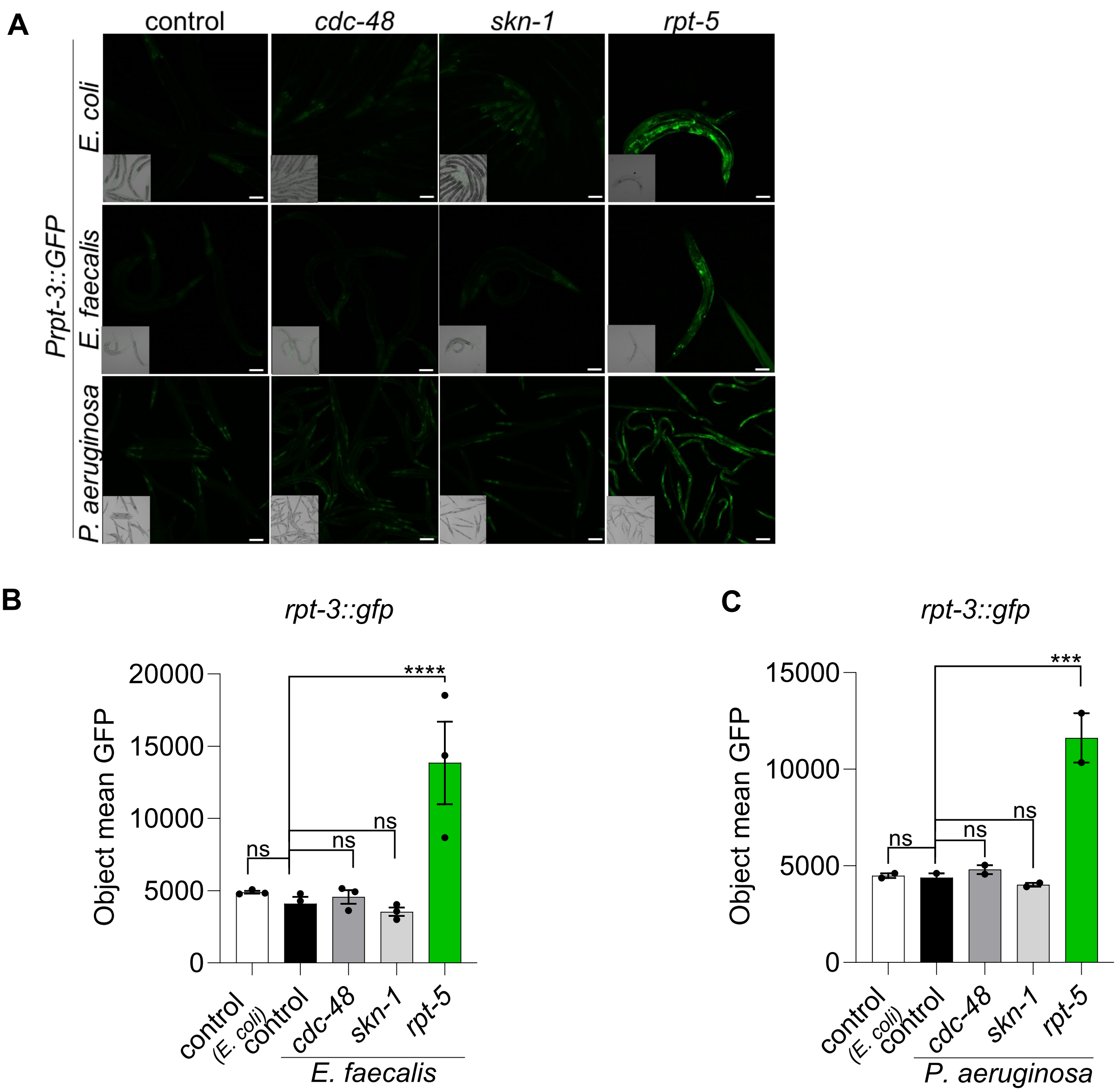

Figure S2. SKN-1A gene expression is not triggered by exposure to pathogen (A) Worms containing a reporter of SKN-1A activity, the *Prpt-3::GFP* transgene, were exposed to *E. faecalis* or *P. aeruginosa*. *Prpt-3::GFP* levels were observed by fluorescence microscopy. No signal was observed under the evaluated conditions, except in the *rpt-5* RNAi positive control. (B, C) Quantification of GFP fluorescence in animals with *Prpt-3::GFP* expression reporter, shown in panel (A), was performed using a Cytation 5 imaging plate reader. The y-axis represents the mean pixel count measured in nematodes following pathogen exposure. Analysis was conducted on three and two biological replicates following *E. faecalis* and *P. aeruginosa* exposure, respectively, each with at least 50 worms. Error bars indicate the standard error of the mean (SEM). The asterisks indicate the statistical significance of the bracketed comparisons.: $*p < 0.05$; $**p < 0.01$; $***p < 0.001$; $****p < 0.0001$; ns, not significant.

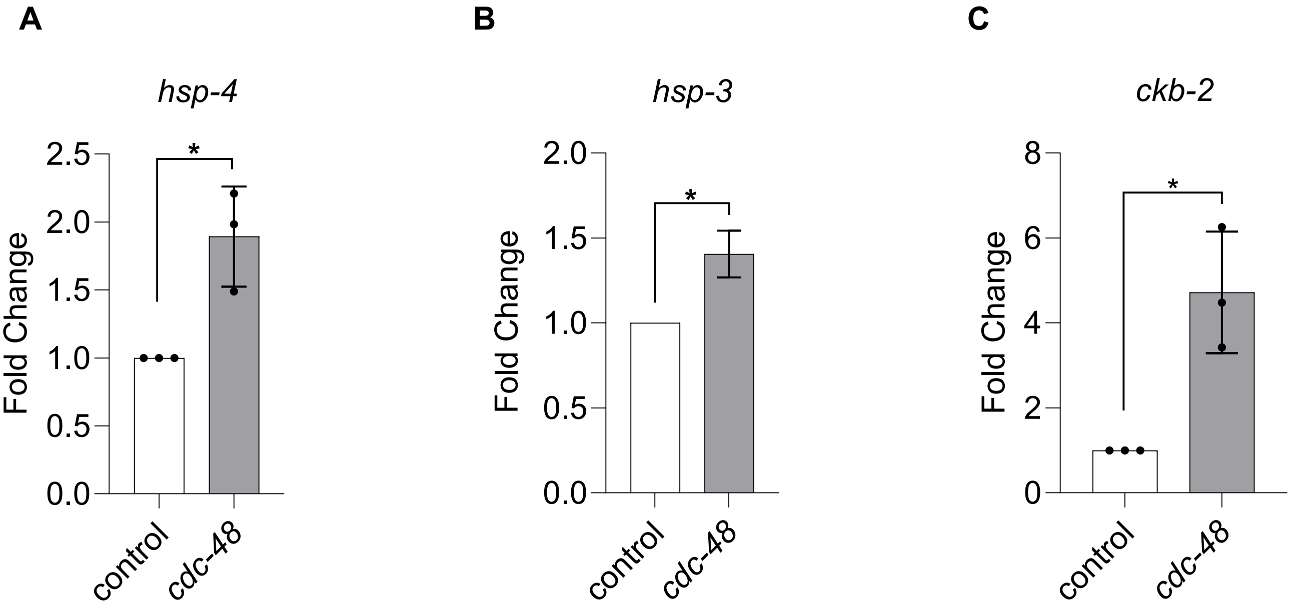

Figure S3. Loss of CDC-48 activates the expression of XBP-1 regulated genes
qRT-PCR quantification analysis of *hsp-4* (A), *hsp-3* (B), and *ckb-2* (C) in wild-type N2 worms exposed to control and *cdc-48* RNAi and retained on *E. coli*. Gene expression values were normalized to the *act-1* housekeeping gene and compared to the control RNAi animals set to one. Error bars represent the SEM of the biological replicates. The statistical significance as compared to the control is denoted as $*p < 0.05$.

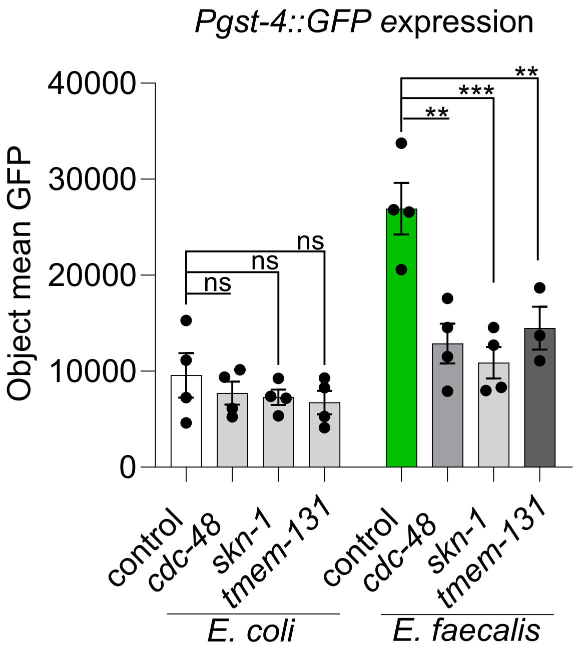

Figure S4. The oxidative stress response is not triggered under ER stress conditions. Quantification of the mean GFP fluorescence of *Pgst-4::gfp* animals with a Cytation 5 imaging plate reader following RNAi of the indicated genes and exposure to *E. coli* or *E. faecalis*. The y-axis represents the mean pixel count measured. Analysis was conducted on three biological replicates, each with at least 50 worms. Error bars indicate the standard error of the mean (SEM). For each bacterial exposure, the asterisks indicate the statistical significance of the bracketed comparisons.. ∗∗$p < 0.01$; ∗∗∗$p < 0.001$; ns, not significant.
